# Combined PD-1 blockade and GITR triggering induce a potent antitumor immunity in murine cancer models and synergizes with chemotherapeutic drugs

**DOI:** 10.1186/1479-5876-12-36

**Published:** 2014-02-07

**Authors:** Lei Lu, Xiaobing Xu, Bin Zhang, Rongsheng Zhang, Hongzan Ji, Xuan Wang

**Affiliations:** 1 Department of Surgical Oncology, Jindu Hospital, Nanjing 210002, China; 2 Department of Gastroenterology and Hepatology, Jinling Hospital, Clinical School of Nanjing, Second Military Medical University, Nanjing 210002, China

**Keywords:** PD-1, GITR, Monoclonal antibody, Immunotherapy

## Abstract

**Background:**

The coinhibitory receptor Programmed Death-1 (PD-1) inhibits effector functions of activated T cells and prevents autoimmunity, however, cancer hijack this pathway to escape from immune attack. The costimulatory receptor glucocorticoid-induced TNFR related protein (GITR) is up-regulated on activated T cells and increases their proliferation, activation and cytokine production. We hypothesize that concomitant PD-1 blockade and GITR triggering would synergistically improve the effector functions of tumor-infiltrating T cells and increase the antitumor immunity. In present study, we evaluated the antitumor effects and mechanisms of combined PD-1 blockade and GITR triggering in a clinically highly relevant murine ID8 ovarian cancer model.

**Methods:**

Mice with 7 days-established peritoneal ID8 ovarian cancer were treated intraperitoneally (i.p.) with either control, anti-PD-1, anti-GITR or anti-PD-1/GITR monoclonal antibody (mAb) and their survival was evaluated; the phenotype and function of tumor-associated immune cells in peritoneal cavity of treated mice was analyzed by flow cytometry, and systemic antigen-specific immune response was evaluated by ELISA and cytotoxicity assay.

**Results:**

Combined anti-PD-1/GITR mAb treatment remarkably inhibited peritoneal ID8 tumor growth with 20% of mice tumor free 90 days after tumor challenge while treatment with either anti-PD-1 or anti-GITR mAb alone exhibited little antitumor effect. The durable antitumor effect was associated with a memory immune response and conferred by CD4^+^ cells and CD8^+^ T cells. The treatment of anti-PD-1/GITR mAb increased the frequencies of interferon-γ-producing effector T cells and decreased immunosuppressive regulatory T cells and myeloid-derived suppressor cells, shifting an immunosuppressive tumor milieu to an immunostimulatory state in peritoneal cavity. In addition, combined treatment of anti-PD-1/GITR mAb mounted an antigen-specific immune response as evidenced by antigen-specific IFN-γ production and cytolytic activity of spleen cells from treated mice. More importantly, combined treatment of anti-PD-1/GITR mAb and chemotherapeutic drugs (cisplatin or paclitaxel) further increased the antitumor efficacy with 80% of mice obtaining tumor-free long-term survival in murine ID8 ovarian cancer and 4 T1 breast cancer models.

**Conclusions:**

Combined anti-PD-1/GITR mAb treatment induces a potent antitumor immunity, which can be further promoted by chemotherapeutic drugs. A combined strategy of anti-PD-1/GITR mAb plus cisplatin or paclitaxel should be considered translation into clinic.

## Background

Epithelial ovarian carcinoma (EOC) is the most lethal gynecological tumor in women, with 22,280 new cases and 15,460 deaths estimated in the United States for 2012 [1]. Five-year overall survival is approximately 45%, and, even with modern surgical and chemotherapeutic strategies, most cases with advanced disease relapse and succumb to the disease [2,3]. Thus, it is major requisite to develop novel strategies for improving the outcomes of ovarian cancer.

Emerging evidence indicates that EOC should be amenable to the immunotherapy [4]. Previous studies show that EOC cells express many tumor-associated antigens against which specific immune responses can be detected [5-9]. The pioneer studies by Coukos and colleagues further indicate immune response in tumor tissue is associated with clinical outcome of patients with EOC as evidenced by the close correlation between patient survival and tumor infiltration with CD3^+^ T cells in the large annotated clinical samples [10]. In addition, in patients with EOC metastases are frequently restricted to the peritoneal cavity where the tumor is directly accessible, obviating the need for systemic delivery of immune-modulatory agents [11]. Despite the abundant evidence supporting EOC immunotherapy, clinical success with immune-based therapies for EOC has generally been modest [12].

Programmed Death 1 (PD-1) protein is a key co-inhibitory receptor which is inducibly expressed on activated T cells, B cells, macrophages, dendritic cells (DC) and monocytes. PD-1 has been shown to inhibit both adaptive and innate immune response when engagement of its ligands PD-L1 (B7-H1) and PD-L2 (B7-DC), which are expressed by tumor cells, stromal cells, or both [13,14]. PD-L1 is the primary PD-1 ligand that is up-regulated in solid tumors, where it can inhibit cytokine production and the cytolytic activity of PD-1^+^ tumor-infiltrating CD4^+^ and CD8^+^ T cells [15,16]. Blockade of PD-1/PD-L1 interaction induces potent antitumor effects in animal models [14,17,18]; furthermore, recent clinical trials show that monoclonal antibodies (mAbs) specific for PD-1 and PD-L1 mount an impressive antitumor effect in several types of solid tumors with complete regression observed in some patients [19-21], demonstrating PD-1/PD-L1 pathway as a highly promising target for cancer immunotherapy.

Glucocorticoid-induced TNFR related protein (GITR; a.k.a. TNFRSF18) is a co-stimulatory molecule of TNF receptor family expressed on activated T cells, B cells, NK and myeloid cells and regulatory T cells (Treg) [22]. As a co-stimulatory molecule, GITR engagement increases proliferation, activation, and cytokine production of CD4^+^ and CD8^+^ T cells [22]. GITR-specific agonistic mAbs or recombinant GITR ligand (GITRL) fusion proteins have been shown to induce tumor regression *in vivo* through the activation of CD4^+^ T cells, CD8^+^ T cells and NK cells in several tumor models [23-25]. In addition, GITR triggering can also abrogate the immunosuppressive activity of natural Treg [26]; however, evidence indicates that the expansion of CD4^+^ effector cells, rather than Treg inhibition, is the primary mechanism underlying the antitumor effects mediated by GITR-targeting mAbs [27]. A humanized GITR-targeting mAb (TRX518) is currently being evaluated in Phase I clinical trials treating patients with late-stage melanoma [28].

Although antagonist PD-1 or agonistic GITR mAbs can promote the rejection of some murine tumors, however, poorly immunogenic tumors such as ID8 ovarian cancer do not respond to single immunomodulating mAb therapy [29]. We hypothesized that combined PD-1 blockade and GITR triggering could synergistically potentiate the antitumor immune response. In this study, using ID8 murine ovarian cancer model, we evaluated the antitumor effects and underlying mechanisms of combined anti-PD-1/GITR mAb treatment.

## Methods

### Mice

Female C57BL (6–8 week old) were purchased from the Model Animal Research Center of Nanjing University. Animal use was approved by the Institutional Review Board of Jindu Hospital, Nanjing, China.

### Cell culture

ID8, a clone of the MOSEC ovarian carcinoma of C57BL/6 origin was a gift from Dr. George Coukos (University of Pennsylvania, Philadelphia, USA). Murine 4 T1 breast cancer cells (BALB/c background) and T cell lymphoma EL4 cells (C57BL/6 background) were kindly sent by Dr. Pu Liu (University of Washington, WA, USA). Tumor cells were cultured in the complete DMEM medium supplemented with 10% FBS (Thermo Scientific, Rockford, IL), 100 U/mL penicillin and 100 μg/mL streptomycin before cell suspensions were prepared and transplanted to mice. The EL4 cells and splenocytes were maintained in a complete medium of RPMI-1640 supplemented with 10% FBS, 25 mM HEPES, 2 mM glutamine, 100 U/mL penicillin and 100 μg/mL streptomycin.

### Reagents

Therapeutic anti-GITR (Clone DTA-1; Catalog#:BE0063), anti-PD-1 (Clone RMP1-14; Catalog#BE0146), anti-CD4 (Clone GK1.5; Catalog#:BE0003-1), anti-CD8 (Clone 2.43; Catalog#:BE0061), anti-NK1.1 (Clone PK136; Catalog#:BE0036) and control rat IgG (Clone 2A3; Catalog#:BE0089) monoclonal antibodies (mAb) were purchased from BioXcell (West Lebanon, NH). Antibodies used for flow cytometry were purchased from Tianjing Sungene (eBioscience, San Diego, CA) and eBioscience (San Diego, CA). H-2Db-restricted mesothelin-derived (MESO406-414: GQKMNAQAI) or control lymphocytic choriomeningitis virus (LCMV) glycoprotein (GP)-derived (GP33-41: KAVYNFATC) epitope peptide were synthesized by GenScript (Beijing, China) and more than 95% of purity were confirmed by HPLC. Peptides were reconstituted in DMSO with final concentration of 10 mg/mL.

### Animal experiments

Mice (10 mice/group) were transplanted intraperitoneally (i.p.) with 5 × 10^6^ ID8 cells in 0.1 mL of PBS on day 1. On days 8, 11 and 15, mice received the i.p. injection of 250 μg of control, anti-PD-1, anti-GITR or anti-PD-1/GITR mAb in 250 μL of PBS. For combined mAb/cisplatin or paclitaxel therapy experiments, mice (10 mice/group) bearing 8 days established ID8 ovarian cancer were first pretreated with a dose of cisplatin (10 mg/kg in 100 μL PBS) or paclitaxel (10 mg/kg in 100 μL PBS) followed by three doses of control or anti-PD-1/GITR mAb at the schedule described above starting on day 9. Long-term surviving mice from combined anti-PD-1/GITR treatment were rechallenged i.p. with 5 × 10^6^ ID8 cells The mice were weighed every other day and checked for the clinical sign of swollen bellies indicative of ascites formation and for the evidence of toxicity such as respiratory distress, mobility, weight loss, diarrhea, hunched posture, and failure to eat while histopathology was conducted on major organs (i.e., liver, kidney, intestines, lungs, and colon). Mice were euthanized when they developed ascites and had a weight increase > 30% of original weight on day 1. For combined therapy experiments in the 4 T1 breast cancer model, mice (5/group) were transplanted subcutaneously (s.c.) with 5 × 10^5^ 4 T1 cells in 0.1 mL of PBS on day 1. On days 8, mice were intratumorally (i.t.) treated with cispaltin or paclitaxel followed by mAb using the dose/schedule described in ID8 model. Two perpendicular diameters of s.c. tumors were measured every other day using a caliper and tumor volumes were calculated according to the formula: 1/2 × (length) × (width)^2^. Mice were sacrificed when they seemed moribund or their tumors reached 10 mm in diameter. The survival of mice was recorded and overall survival was calculated. For lymphocyte depleting experiments, mice were injected i.p. with 500 μg of mAb against CD8, CD4, or NK1.1, 1 day before and two days after tumor challenge, followed by injection of 250 μg every 5 days throughout the experiment. The efficacy of cell depletion was verified by flow cytometric analysis of lymphocyte subsets in peripheral blood (data not shown).

### Evaluation of tumor-associated immune cells (TAC)

Tumor-bearing mice were euthanized 7 days after the last treatment described as in animal experiments. To obtain TAC, 3 ml PBS was injected into the peritoneal cavity of mice with ID8 tumors immediately after euthanasia, their belly was massaged and the fluid was removed, filtered through a 70 μM cell strainer (BD Biosciences), washed and resultant peritoneal cells (including immune cells and tumor cells) were subjected to further analysis.

For flow cytometric staining, single cell suspensions of peritoneal cells were washed with FACS staining buffer and incubated with mouse Fc receptor binding inhibitor (eBioscience) for 10 minutes before staining with mAbs (eBioscience) against mouse CD45 (clone 30-F11), CD3 (clone 145-2C11), CD4 (clone GK1.5), CD8 (clone 53–6.7), CD19 (clone eBio1D3), CD11b (clone M1/70), Gr-1 (clone RB6-8C5), CD44 (clone 1 M7) and CD62L (clone MEL-14) for 30 minutes. For intracellular staining of FoxP3 (clone FJK-16 s), cells were fixed, permeabilized, and stained following the instruction of Cytofix/Cytoperm kit (BD Bioscience). For intracellular staining of IFN-γ (clone XMG1.2), cells were restimulated *in vitro* with 50 ng/ml PMA and 1 μg/ml ionomycin for 4 hours prior to the analysis of IFN-γ secretion in CD4^+^ or CD8^+^ subsets. Flow cytometry was performed using FACSCalibur (BD Biosciences) and the lymphocyte population was selected by gating CD45-positive cells. The data were analyzed using Flow Jo software (Tree Star, Ashland, OR). All flow cytometry experiments were performed at least 3 times.

### Evaluation of antigen-specific immune response

Isolated splenocytes from treated mice were cultured in the presence of 10 μg/mL H-2Db-restricted mesothelin-derived (MESO406-414) or LCMV-GP (GP33-41) epitope peptide for 3 days. IFN-γ concentration in the supernatants was determined by Mouse IFN-γ Quantikine ELISA Kit (R&D systems).

For mesothelin-specific CTL assays, effector cells were obtained by coculturing 5 × 10^6^ splenocytes with 5 × 10^5^ UV-irradiated ID8 cells for 4 days. Peptide-pulsed EL4 target cells were generated by adding 10 μg/ml of peptide and incubating for 4 hours. CTL activity was measured using the CytoTox96 Non-Radioactive Cytotoxicity Assay kit (Promega, Madison, WI) following the manufacturer’s instructions. In brief, target cells were incubated with varying numbers of effector cells for about 4 hours, and supernatants were then analyzed for lactate dehydrogenase release. The results are expressed as percent specific lysis, calculated as (Experimental release-Spontaneous release/Total release-Spontaneous release) × 100.

### Statistics

Results were expressed as mean ± SEM. All statistical analyses were performed using GraphPad Prism 5. Student’s t test was used to compare the statistical difference between two groups and one-way ANOVA was used to compare three or more groups. Survival rates were analyzed using the Kaplan–Meier method and evaluated with the log-rank test with Bonferroni correction. Significant differences were accepted at p < 0.05.

## Results

### Combined anti-PD-1/GITR mAb treatment induced potent antitumor effects in ID8 ovarian cancer

**Figure 1:**
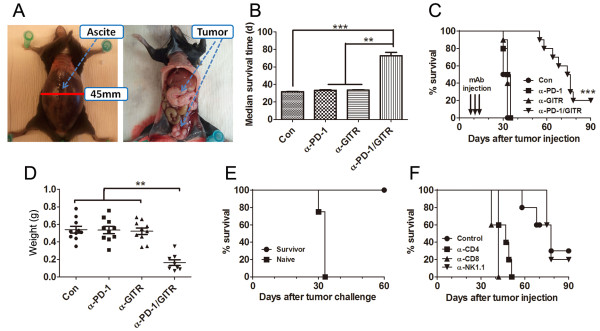
**Treatment of combined anti-PD-1/GITR mAb induced tumor-specific long-lasting immunity against ID8 ovarian cancer. A**, The typical presentation of ID8 ovarian cancer in C57BL/6 mice. The left and right picture shows the macroscopic appearance of ascites and ID8 tumor mass in peritoneal cavity of mice respectively. **B**, Mice (10 mice/group) were transplanted i.p. with 5 × 10^6^ ID8 cells on day 1 and treated with 250 μg of control, anti-PD-1, anti-GITR and anti-PD-1/GITR mAb on day 8, 11 and 15. Mean survival time of tumor-bearing mice was calculated. **C**, Overall survival of mice was recorded. **D**, The peritoneal tumor masses were weighed when mice were euthanized with each dot representing each mouse. **E**, Long-term surviving (90 days after first tumor challenge) mice from combined mAb treatment group were rechallenged with ID8 cells and their overall survival was recorded. Naïve mice were challenged with ID8 cells as control. **F**, Mice (5 mice/group) treated with combined anti-PD-1/GITR mAb were also injected with an anti-CD4, anti-CD8, anti-NK1.1, or control mAb with 500 μg of each mAb per mouse 1 day before and two days after tumor challenge followed by injection of 200 μg every 5 days thereafter for the duration of the experiments and their overall survival were recorded. Data are representative of 2 independent experiments for B-E. **P < 0.05, ***P < 0.001, combined mAb vs control or single mAb.

We first assessed the antitumor effects of either single or combined anti-PD-1/GITR mAb in murine ID8 ovarian cancer, a highly clinical relevant model with ascites formation and metastases in peritoneal cavity (Figure 1A). Group of C57BL/6 mice were i.p. transplanted with 5 × 10^6^ ID8 cells on day 1 and then were treated with i.p. injection of control, anti-PD-1, anti-GITR or anti-PD-1/GITR mAb on day 8, 11 and 15. Control mAb-treated mice developed ascites about 30 days after tumor challenge and had to be sacrificed. Although either single anti-PD-1 or anti-GITR mAb exhibited little antitumor effect, combined anti-PD-1/GITR mAb treatment significantly prolonged overall survival time of mice (Figure 1B; median survival time 31.50, 34.00, 33.00 or >75.00 days for control, anti-PD-1, anti-GITR or anti-PD-1/GITR mAb group respectively; p < 0.01, combined mAb compared to single or control mAb) with 20% (2 out of 10 mice) of mice remaining tumor-free (confirmed by laparotomy) 90 days after tumor challenge (Figure 1C). The weight of tumor masses from mice treated with combined mAb also greatly decreased compared with that from control or single mAb treated mice (Figure 1D). A repeat of the experiment gave similar results (data not shown). Long-term surviving mice from first tumor challenge (90 days after first challenge), but not naïve mice, were resistant to a subsequent rechallenge i.p. with ID8 cells (Figure 1E), indicating that combined anti-PD-1/GITR mAb treatment mounted an antitumor memory immune response in mice. Cell depleting experiments showed that tumor protection was dependent on the CD4^+^ and CD8^+^ T cells as removal of CD4^+^ or CD8^+^ T cells abrogated the antitumor effect conferred by anti-PD-1/GITR mAb treatment (Figure 1F).

### Combined anti-PD-1/GITR mAb treatment shifted an immunosuppressive to an immunostimulatory tumor milieu

**Figure 2:**
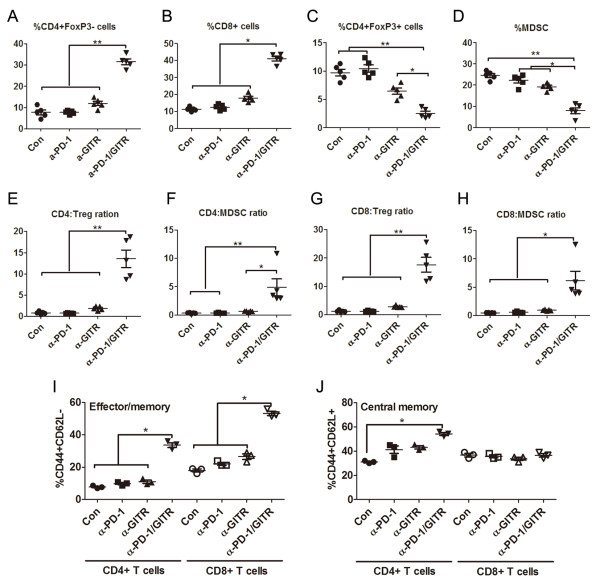
**Subpopulation analysis of tumor-associated immune cells (TAC) from treated mice.** Mice (5 mice/group) were transplanted i.p. with 5 × 10^6^ ID8 cells on day 1 and treated with 250 μg of control, anti-PD-1, anti-GITR and anti-PD-1/GITR mAb on day 8, 11 and 15. Seven days after last mAb injection, TAC from treated mice was analyzed by flow cytometry for various immune cell subpopulations. The percentages of CD4^+^FoxP3^-^, CD8^+^, CD4^+^FoxP3^+^ Treg and CD11b^+^GR-1^+^ MDSC in TAC are shown in **A, B, C** and **D** respectively. The ratios of both CD4^+^ and CD8^+^ T cells to Treg and MDSC are shown in **E, F, G** and **H** respectively. The frequencies of CD44^+^CD62L^-^ effector/memory and CD44^+^CD62L^+^ central memory in CD4^+^ and CD8^+^ T cells from TAC are shown in **I** and **J** respectively. Each dot represents the data from each mouse and Data are representative of 2 independent experiments, *P < 0.05, **P < 0.01.

To define the immune mechanisms of the synergistic antitumor effects of combined PD-1 blockade and GITR triggering, we analyzed the phenotype and function of tumor-associated immune cells (TAC) harvested from peritoneal cavity of treated mice 7 days after last mAb injection. Compared with control or single mAb, combined mAb significantly increased the percentages of effector CD4^+^FoxP3^-^ T cells (mean value 7.70%, 7.78%, 11.94% and 31.50% for control, anti-PD-1, anti-GITR and anti-PD-1/GITR group respectively; p < 0.01) and CD8^+^ T cells (mean value 11.34%, 12.62%, 17.96% and 41.04%; p < 0.05) and decreased the frequency of CD4^+^FoxP3^+^ regulatory T cells (Treg; mean value 9.72%, 10.44%, 6.44% and 2.54%; p < 0.05 and p < 0.01 compared to anti-GITR and control or anti-PD-1 respectively) and CD11b^+^GR-1^+^ myeloid-derived suppressor cells (MDSC; mean value 24.58%, 22.28%, 19.18% and 8.04%; p < 0.05 and p < 0.01 compared to anti-GITR or anti-PD-1 and control respectively) in TAC on day 7 after treatment (Figure 2A-D); These contrasting changes in effector and immunosuppressive cells gave rise to the significantly elevated ratios of both effector CD4^+^ and CD8^+^ T cells to Treg and MDSC in peritoneal cavity of mice receiving combined mAb treatment (Figure 2E-H). With regards to individual mAb treatment, GITR engagement modestly elevated the percentage of effector CD4^+^FoxP3^-^ T cells and CD8^+^ T cells and attenuated the frequencies of Treg and MDSC; however, single PD-1 had little effect on these subsets. We also noted an increase in absolute number of total peritoneal immune cells from 2 mAb treated mice (mean value (× 10^6^/mouse): 3.8, 3.9, 4.2 and 6.5 for control, anti-PD-1, anti-GITR and anti-PD-1/GITR group respectively; p < 0.05) and the changes in absolute number of each subset had a similar trend to their percentages (data not shown).

**Figure 3:**
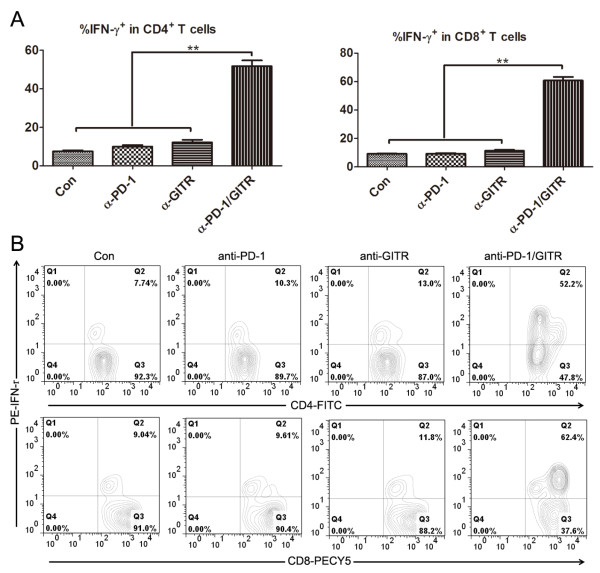
**Functional analysis of IFN-γ production in tumor-associated CD4**^**+**^**and CD8**^**+**^**T cells from treated mice.** Mice (5 mice/group) were transplanted i.p. with 5 × 10^6^ ID8 cells on day 1 and treated with 250 μg of control, anti-PD-1, anti-GITR and anti-PD-1/GITR mAb on day 8, 11 and 15. Seven days after last mAb injection, tumor-associated CD4^+^ and CD8^+^ T cells from peritoneal cavity of treated mice were dissected of IFN-γ production by intracellular cytokine staining. The frequencies of IFN-γ-producing cells in tumor-associated CD4^+^ and CD8^+^ T cells are shown in **A**. The representative dotplots are shown in **B** with upper and bottom panels displaying IFN-γ staining in gated CD4^+^ and CD8^+^ T cells respectively. Data are representative of 2 independent experiments, **P < 0.01.

Analysis of CD44 and CD62L expression on CD4^+^ and CD8^+^ T cells demonstrated that TAC from anti-PD-1/GITR mAb treated mice contained significantly increased percentage of CD44^+^CD62L^-^ effector/memory cells (mean value 7.77%, 9.83%, 11.00% and 33.70% for CD4 cells; 17.93%, 22.10%, 26.50% and 53.30% for CD8 cells; p < 0.05 for both cells) and CD44^+^CD62L^+^ central memory cells (31.00%, 41.20%, 43.13% and 54.17% for CD4 cells; 36.67% , 35.67%, 33.67% and 36.57% for CD8 cells; p < 0.05 for CD4 cells) compared with that from control or single mAb treated mice (Figure 3I, J), where we could see more effector/memory cells or central memory cells in respective CD8^+^ or CD4^+^ T cells from combination treatment.

Further functional analysis showed that significantly elevated frequencies of IFN-γ-producing cells were seen in tumor-associated CD4^+^ and CD8^+^ T cells from combined mAb treated mice (Figure 3A). The representative dotplots were shown in Figure 3B.

Together, the data indicate that PD-1 blockade and GITR triggering synergistically creates higher ratios of effector T cells to immunosuppressive cells in peritoneal cavity of treated mice, which represents the shift of an immunosuppressive tumor milieu to an immunostimulatory state which is more permissive for immune mediated tumor destruction.

### Combined anti-PD-1/GITR mAb treatment mounted an antigen-specific CTL response

**Figure 4:**
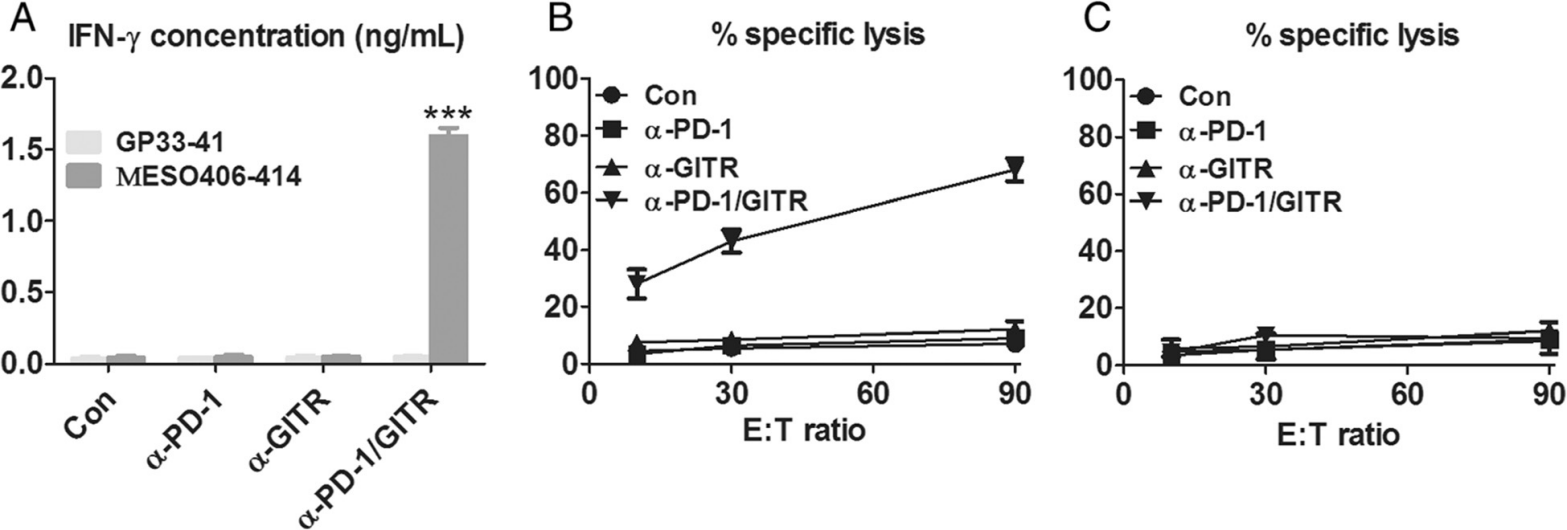
**Treatment with anti-PD-1/GITR mAb induced a tumor antigen-specific CTL response in treated mice. A**, Mice (5 mice/group) were transplanted i.p. with 5 × 10^6^ ID8 cells on day 1 and treated with 250 μg of control, anti-PD-1, anti-GITR and anti-PD-1/GITR mAb on day 8, 11 and 15. Seven days after last mAb injection, splenocytes from treated mice were cultured in the presence or absence of H-2Db-restricted mesothelin or control GP33-41 epitope peptide for 3 days and IFN-γ production in the supernatants were determined by ELISA. **B**, Pooled splenocytes (5 × 10^6^) from control or combined anti-PD-1/GITR mAb were incubated with 5 × 10^5^ UV-irradiated ID8 cells for 4 days prior to subject to analysis of antigen-specific CTL activity by CytoTox 96 Non-radioactive cytotoxicity assay using EL4 cells pulsed with H-2Db-restricted mesothelin as target cells. **C**, As a specific control, pooled splenocytes were tested cytotoxicity against EL4 target cells pulsed with control H-2Db-restricted GP33-41 peptide.

We next evaluated the antigen-specific immune response in treated mice. As ID8 cancer cells express the mesothelin, a well-known tumor antigen [29,30], we harvested splenocytes from treated mice, and cultured them in the presence of 10 μg/mL of H-2Db-restricted mesothelin-derived epitope peptide (MESO406–414) or control GP33–41 epitope peptide for 3 days and assayed IFN-γ secretion in culture supernatants by ELISA. As shown in Figure 4A, splenocytes from combined mAb-treated mice produced significantly higher levels of IFN-γ when stimulated by the mesothelin epitope peptide compared to control or single mAb-treated mice (P < 0.01). No IFN-γ secretion were seen in the culture supernatants of splenocytes from treated mice upon stimulation by control GP33–41 epitope peptide, suggesting the elicitation of mesothelin-specific immune response in combined mAb treated mice. We further evaluated the antigen-specific killing activity by splenocytes from treated mice. Splenocytes were restimulated with UV-irradiated ID8 cells for 5 days before CTL assays were performed using EL4 cells pulsed with MESO406–414 or GP33–41 epitope peptide as target cells. As shown in Figure 4B and 4C, splenocytes from anti-PD-1/GITR treated mice, but not control or single mAb treated mice, exhibited a prominent cytotoxicity against EL4 cell pulsed with MESO406–414 but not GP33–41 epitope peptide, suggesting the induction of mesothelin-specific CTL response in combined mAb treated mice.

### Combined treatment of anti-PD-1/GITR mAb and chemotherapeutic drugs induced a durable antitumor effect

**Figure 5:**
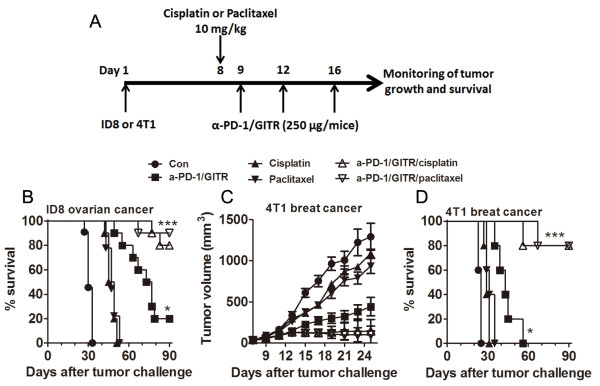
**Combined treatment of anti-PD-1/CD137 mAb and chemotherapeutic drugs induced complete remission of established tumors. A**, The experimental schematic: mice (10 mice/group) were transplanted i.p. with 5 × 10^6^ ID8 cells **(B)** or s.c. with 5 × 10^5^ 4 T1 cells **(C and D)** on day 1 and were pretreated with a dose of cisplatin (10 mg/kg) or paclitaxel (10 mg/kg) on day 8 and followed by three doses of anti-PD-1/GITR mAb (250 μg/mice) on day 9, 12 and 16. The mice were treated with PBS, cisplatin, paclitaxel or anti-PD-1/GITR mAb alone as controls. **B**, The overall survival of mice in the ID8 cancer model. **C**, The tumor growth curve in the 4 T1 breast cancer model. **D**, The overall survival of mice in the 4 T1 breast cancer model. Data are representative of 2 independent experiments. *P < 0.05, ***P < 0.001, compared to control-treated mice.

To mimic clinical application more closely, we evaluated whether anti-PD-1/GITR mAb could synergize with cisplatin and paclitaxel, two commonly used chemotherapeutic drugs for advanced EOC, to produce durable antitumor effects. We treated ID8 tumor-bearing mice with i.p. injection of a dose of cisplatin or paclitaxel 1 day before three doses of anti-PD-1/GITR mAb within a week (Figure 5A). Similar to the result in Figure 1C, combined anti-PD-1/GITR mAb treatment significantly prolonged the survival of mice with 20% of mice remaining tumor-free 90 days after tumor challenge, and cisplatin or paclitaxel pretreatment alone also modestly increased the survival of mice; strikingly, combined treatment of anti-PD-1/GITR mAb plus cisplatin or paclitaxel produced an impressing antitumor effect, resulting in the long-term survival of more than 80% mice (8 or 9 mice out of 10 for anti-PD-1/GITR/cisplatin or anti-PD-1/GITR/paclitaxel respectively) at the terminate of experiments (Figure 5B). With regard to treatments combining single mAb with chemotherapeutic drug (anti-PD-1/cisplatin, anti-PD-1/paclitaxel, anti-GITR/cisplatin, or anti-GITR/paclitaxel), we observed a similar antitumor effect as treatment with combined anti-PD-1/GITR mAb alone (data not shown).

To validate the above results, we repeated an experiment with the 4 T1 breast cancer model. BALB/c mice with s.c. established 4 T1 tumors (4-6 mm in diameter) were injected i.t. with anti-PD-1/GITR mAb, cisplatin, paclitaxel or a combination of mAb and cisplatin or paclitaxel, using the same dose/schedule as in the ID8 experiment. As shown in Figure 5B and 5C, combined anti-PD-1/GITR mAb exhibited a similar antitumor effect in this model with evident prolongation of overall survival (median survival 43 vs 25 days, p < 0.05 compared with control), however, all mice succumbed to tumor growth and died by 60 days after tumor challenge. A dose of cisplatin or paclitaxel alone exhibited a mild antitumor effect (median survival 29 or 31 vs 25 days). In contrast, a combination of anti-PD-1/GITR mAb with cisplatin or paclitaxel significantly suppressed tumor growth and 80% of mice (4 mice out of 5) survived tumor-free when the experiment was terminated 90 days after tumor challenge. These long-term surviving mice developed a systemic immune response with memory as demonstrated by their resistance to rechallenge with 4 T1 cells (data not shown).

## Discussion

The antitumor effect of immunotherapy remains insufficient to achieve long-lasting clinical responses in patients with advanced EOC. In this study, we show that combined anti-PD-1/GITR mAb elicited a potent antitumor immune response resulting in significant tumor growth suppression in a highly clinically relevant ovarian cancer model; more importantly, anti-PD-1/GITR mAb show a clearly synergistic antitumor effect with cisplatin and paclitaxel, two most commonly used chemotherapeutic drugs for EOC patients, in murine ID8 ovarian cancer (C57BL/6 origin) and 4 T1 breast cancer (BALB/c origin) models with two strains of mice with different genetic backgrounds. Our findings provide a strong rationale for translation of this combination strategy to clinic.

We defined the immune mechanisms of the therapeutic effects by combined anti-PD-1/GITR mAb. Analyzing the components of TAC from treated mice, we found that single GITR triggering slightly increased or decreased the percentages of CD4^+^ and CD8^+^ T cells or Treg and MDSC respectively in this tumor model, which is consistent with previous reporting effects of GITR triggering on these subsets [31,32]; although single PD-1 blockade had little effect on the components of TAC, combining PD-1 blockade with GITR activation significantly promoted the accumulation of CD4^+^ and CD8^+^ T cells with concomitant attenuation of Treg and MDSC, giving rise to a favorable ratio of the effector T cells to the immunosuppressive cells which is closely correlated with the effective immunotherapy as previously stated [33,34]. Furthermore, increased frequencies of CD44^+^CD62L^-^ effector/memory and/or CD44^+^CD62L^+^ central memory cells were present in CD4^+^ and CD8^+^ T cells from combined mAb treated mice and these effector or memory T cells produced much more IFN-γ in response to TCR engagement. Consistent with these observations, CD4^+^ and CD8^+^ T cells were indispensable for tumor protection conferred by combined treatment as shown in cell depletion experiments and long-lasting memory immune response was developed as evidence by the resistance to tumor rechallenge. The data support that combined PD-1 blockade and GITR triggering shifted an immunosuppressive tumor environment to an immunostimulatory state, which favorably contributes to a durable antitumor effect.

We detected a systemic antigen-specific CTL response to ID8-expressing mesothelin in anti-PD-1/GITR mAb treated mice, as evidenced by mesothelin-specific IFN-γ production and cytolysis by CD8^+^ T cells from these mice. We also observed an increased percentage of splenic CD8^+^ T cells in combined mAb-treated mice compared with that in control or single mAb-treated mice (9.3 ± 3.1%, 10.3 ± 2.9%, 10.6 ± 3.0% or 13.1 ± 2.6% for control, anti-PD-1 or anti-OX40 or anti-PD-1/OX40 group); after normalization to the percentage of splenic CD8^+^ T cells, a significantly increased mesothelin-specific IFN-γ production from combined mAb-treated mice was still seen (data not shown). As an endogenous non-mutated antigen, mesothelin should be naturally tolerized against; therefore, the induction of mesothelin-specific CTL response by anti-PD-1/GITR mAb treatment indicates that endogenous tolerance to mesothelin was broken, which is consistent with previous studies demonstrating the overcome of tolerance/ignorance by GITR activation in murine tumors and the presence of mesothelin-specific immune response in patients with cancers expressing high level of mesothelin [24,35,36]. We did not detect mesothelin-specific antibodies in sera from mice treated with combined mAb (data not shown).

Currently, it remains unclear for the mechanisms underlying the synergy between anti-PD-1 and anti-GITR mAb. The insensitivity of ID8 ovarian cancer to treatment of single anti-PD-1 or anti-GITR mAb is in part due to lack of expression of PD-1 ligands PD-L1/2 or GITR ligand GITRL since ligand expression on the tumor has been reported to be correlated with the response to these mAbs [19,37]. We checked the PL-L1 expression on ID8 tumor cells after *in vivo* treatment by anti-GITR mAb and found *de novo* PL-L1 expression on these tumor cells (Additional file 1: Figure S1), which may be partially responsible for the resistance of ID8 tumors to treatment with single anti-GITR mAb and provide a rationale for the synergistic effect of PD-1 blockade and GITR activation in ID8 tumor inhibition. In addition, GITR triggering may attenuate the Treg-mediated suppression of antitumor immunity [32] while PD-1 blockade can release the brake of negative signaling on effector functions of preexisting tumor-associated T cells. By incorporating those two stings of power, combined treatment of anti-PD-1/GITR mAb can mount a potent antitumor immunity. Clearly, more studies are warranted to define further the synergistic mechanisms in this scenario.

Importantly, addition to the anti-PD-1/GITR mAb combination of cisplatin or paclitaxel, two commonly used chemotherapeutic drugs for EOC treatment [38], administered at a dose equivalent to those used clinically [39], provided long term remission in more than 80% of the treated mice. This therapeutic effect was confirmed in the murine 4 T1 breast tumor model with long-term survival 4 of 5 mice receiving combined mAb/cisplatin or paclitaxel treatment. Interestingly, combination of either anti-PD-1 or anti-GITR mAb and chemotherapeutic drugs did not eradicate most of tumor, underscoring the necessity of synergistic PD-1 blockade and GITR triggering in eliciting an optimal antitumor effects. The detailed mechanisms of synergy between cisplatin/paclitaxel and combined immunotherapy remain unclear; however, increasing tumor antigenicity and/or further deleting immunosuppressive cells by chemotherapeutic drugs may be at least partially responsible for their synergism [40]. Future work is needed to elucidate the exact mechanism of action in this context.

With regard to GITR activation, a clinical grade of anti-GITR agonistic mAb is being tested in a phase I clinical trial [28], and GITRL fusion proteins have also shown a promising antitumor potential in preclinical tumor models [25]. In the aspect of blocking the inhibitory PD-1 pathway, PD-1- or PD-L1-targeting antagonistic mAbs have displayed an impressive antitumor effect for the treatment of advanced solid tumors with manageable autoimmune adverse effects [19,21]. It is noteworthy that consequential peritoneal administration of chemotherapeutic drugs and mAbs, a procedure that can be applied clinically in patients with ovarian cancer, did not induce any obvious toxicity such as weight or hair loss in this study. Therefore, our findings provide a strong rationale for clinical trials testing chemo-immunotherapy of ovarian cancer by combined PD-1 blockade using PD-1- or PD-L1-targeting mAb and GITR activation using GITR-specific mAb or GITRL fusion protein and chemotherapeutic drugs.

## Conclusions

To our knowledge, this is the first study showing that combinatorial PD-1 blockade by an antagonistic mAb and GITR triggering by an agonistic mAb induce a potent antitumor effect, which further synergizes with commonly used chemotherapeutic drugs in two murine tumor models. Our demonstration of a synergistic antitumor effect of combining anti-PD-1/GITR mAbs with chemotherapeutic drugs should stimulate further studies to assess the safety and efficacy of similar combinatorial strategies towards ߢtranslationߣ to the clinic.

## Abbreviations

mAb: Monoclonal antibody; TNFR: Tumor necrosis factor receptor; CTL: Cytotoxicity T lymphocytes; Treg: Regulatory T cells; MDSC: Myeloid-derived suppressor cells; EOC: Epithelial ovarian carcinoma; IFN-γ: Interferon-gamma; PD-1: Programmed death protein 1; GITR: Glucocorticoid-induced TNFR related protein; DMEM: Dulbecco’s minimum essential medium; RPMI: Roswell Park Memorial Institute; NK: Natural killer; DC: Dendritic cells; PBS: Phosphate-buffered saline; FBS: Fetal bovine serum; RT-PCR: Reverse transcription polymerase chain reaction; GAPDH: Glyceraldehyde phosphate dehydrogenase; SEM: Standard error of mean.

## Competing interests

The authors declare that they have no competing interests.

## Authors’ contributions

LL performed most of the experiments and drafted the manuscript. XBX carried out the flow cytometric analysis, participated in the design of the study and helped in writing the manuscript. BZ contributed in cell culture techniques and analyzed data. RSZ participated in the statistical analysis and interpretation of data. XW and HZJ conceived and designed the study and critically revised the manuscript. All authors read and approved the final manuscript.

## Supplementary Material

Additional file 1: Figure S1 PD-L1 expression on ex vivo ID8 tumor cells. A, PD-L1 expression on *in vitro* culture ID8 tumor cells was analyzed by flow cytometry using PE conjugated anti-PD-L1 (clone MIH5) or isotype control (clone eBR2a; rat IgG2a) mAb (all from eBioscience). Filled gray line indicates isotype control staining, and red line indicates PL-L1 staining. B, B6 mice were injected i.p. with 5 × 10^6^ ID8 cells and treated with 250 μg of control or anti-GITR mAb on day 8. Two days later, tumor cells were collected and PD-L1 expression was analyzed by flow cytometry as above. Filled gray line indicates isotype control staining, blue line indicates tumors from control-treated mice, and green line indicates tumors from anti-GITR-treated mice. Click here for file
