# Supplementary figures and images for: Combined PD-1 blockade and GITR triggering induce a potent antitumor immunity in murine cancer models and synergizes with chemotherapeutic drugs
Source: J Transl Med. 2014 Feb 7;12:36. doi: 10.1186/1479-5876-12-36 (PMC4104995; doi:10.1186/1479-5876-12-36)

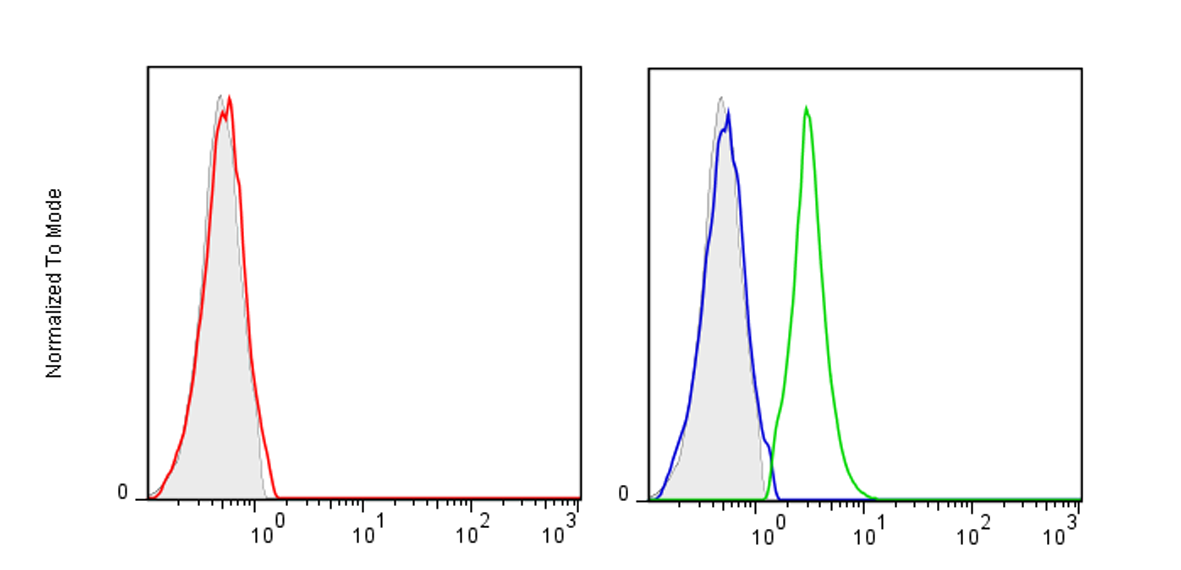

Supplement: Additional file 1: Figure S1 — PD-L1 expression on ex vivo ID8 tumor cells. A, PD-L1 expression on in vitro culture ID8 tumor cells was analyzed by flow cytometry using PE conjugated anti-PD-L1 (clone MIH5) or isotype control (clone eBR2a; rat IgG2a) mAb (all from eBioscience). Filled gray line indicates isotype control staining, and red line indicates PL-L1 staining. B, B6 mice were injected i.p. with 5 × 10^6^ ID8 cells and treated with 250 μg of control or anti-GITR mAb on day 8. Two days later, tumor cells were collected and PD-L1 expression was analyzed by flow cytometry as above. Filled gray line indicates isotype control staining, blue line indicates tumors from control-treated mice, and green line indicates tumors from anti-GITR-treated mice. [file 1479-5876-12-36-S1.tiff]
